# Sarcoidosis as a Great Mimicker: Diagnostic Challenges in a Patient With Coccidioidomycosis

**DOI:** 10.7759/cureus.66716

**Published:** 2024-08-12

**Authors:** Alaa Aldookhi, Mehrnoush Hassas Yeganeh, Wanda Saleh, Manish Adhikari

**Affiliations:** 1 Internal Medicine Residency Program, Capital Health Regional Medical Center, Trenton, USA

**Keywords:** noncaseating granuloma, bronchal alveolar lavage (bal), chest infection, coccidiosis, sarcoidosis

## Abstract

Sarcoidosis presents as a systemic granulomatous disease of unknown etiology, characterized by the development of non-caseating granulomas that commonly affect the lungs, lymph nodes, skin, and eyes. Manifestations of various conditions such as infections, neoplasms, autoimmune, cardiovascular, and drug-induced diseases can bear resemblance to sarcoidosis. Coccidiosis, attributed to protozoan parasites of the Coccidia genus, primarily affects the intestinal tract but may also display systemic symptoms akin to those of sarcoidosis.

In this particular case, we present a 46-year-old immunocompetent gentleman who had an extensive disease despite the patient's well-controlled diabetes and absence of residency in an endemic area with fungal infection, his only pertinent part of the history was his travel to endemic areas for short periods that raised the possibility of thinking about the disseminated fungal infection. The patient's symptoms initially attributed to and treated as sarcoidosis, which later did not respond to steroids, led us to consider other potential causes, including systemic fungal infection

Misdiagnosis of the sarcoidosis bears the risk of inappropriate treatment, potentially leading to exacerbated patient outcomes. Consequently, it is imperative for healthcare providers, particularly rheumatologists, to augment vigilance and conduct comprehensive diagnostic assessments encompassing microbiological testing and histopathological examination.

## Introduction

Sarcoidosis is a disease characterized by the formation of granulomas, and its cause is unknown. It can appear without any symptoms or present life-threatening risks. Nearly half of people with sarcoidosis do not experience any symptoms. Common symptoms of Sarcoidosis include persistent dry cough, fatigue, dyspnea, and erythema nodosum. The diagnosis relies on a thorough medical history, granuloma manifestations in at least two different structures, and the results of staining and negative culture for acid-fast bacilli (AFB). Furthermore, it necessitates the absence of occupational or internal exposure to toxins and the lack of drug-related illnesses. The disease is often diagnosed through chest X-ray findings when investigating other conditions since it frequently affects the lungs, leading to respiratory issues such as shortness of breath and coughing. Chest radiographs often show bilateral hilar lymphadenopathy and interstitial lung disease. About 30%-80% of patients with sarcoidosis have an elevated serum ACE level, with a sensitivity of 22%-86% and a specificity of 54%-95% [1]. the ACE is produced by epithelial granuloma cells, and its serum level reflects the overall granulomatous activity in the body, but there are a large number of false negative results that lead to incorrect management in 23% of patients based on serum ACE level [2]. Clinical severe symptoms, such as lung sarcoidosis that does not respond to treatment, cardiac sarcoidosis, neurosarcoidosis, and sarcoidosis affecting multiple organs, usually have a poor prognosis and are often diagnosed in the late stages of the disease, with limited response to treatment. Effective management, including early diagnosis, treatment, and patient monitoring, has lowered mortality rates [3].

Coccidioides sp. (C. immitis and C. posadasii) is also known as valley fever, desert rheumatism, or San Joaquin Valley fever. These fungi are commonly found in the desert regions of the western hemisphere, including the southwestern United States (central valley of California, Arizona, parts of New Mexico, and Texas), northern Mexico, and certain parts of Central and South America. The infection occurs when people inhale the fungal spores, which are present in the soil of these endemic areas and can become airborne when the soil is disturbed, for example, during construction, farming, windstorms, or earthquakes [4]. People at higher risk of exposure include individuals with specific occupations, such as farming or construction work, as well as those participating in outdoor recreational activities. While most cases are seen in endemic areas, the infection can also be imported to non-endemic areas through travel [5]. The disease can manifest as an asymptomatic infection or as a severe disseminated disease. While most instances of pulmonary coccidioidomycosis resolve spontaneously, individuals with risk factors such as advanced age (over 65 years), diabetes, chronic obstructive pulmonary disease, smoking, or immunosuppression are susceptible to severe pulmonary complications. Extrapulmonary dissemination is infrequent, such as skin, soft tissue, central nervous system, and lymph nodes affecting fewer than 1% of Coccidioides infections, and is more prevalent among immunocompromised individuals [6].

Initial laboratory studies should include a complete blood count, a metabolic panel to include creatinine and liver function testing, calcium levels in blood and urine, vitamin D assays, and an angiotensin-converting enzyme level, testing for the human immunodeficiency virus and tuberculosis should also be performed [7]. further testing may be required to differentiate the cause, while the imaging studies include chest X-ray and computed tomography of the chest, which can show radiological findings of pulmonary sarcoidosis resemble those indicative of coccidioidomycosis or malignancy.

It's important to remember that both conditions can manifest similar respiratory symptoms and radiographic findings, such as hilar lymphadenopathy and pulmonary infiltrates. This case reports a disseminated coccidioidomycosis in an immunocompetent patient with clinical presentations resembling pulmonary sarcoidosis. Flexible bronchoscopy with biopsies, bronchoalveolar lavage (BAL), and pulmonary function testing should be performed in such challenging cases [7]. It is crucial to perform a biopsy and histological examination. Non-caseating granulomas characterize Sarcoidosis, while coccidiosis may reveal organisms within granulomas when special staining is utilized. Accurate identification of coccidia through stool samples, PCR, or serological tests is also helpful to reach an accurate diagnosis. Failure to raise awareness and conduct these tests can result in misdiagnosis as sarcoidosis. Sarcoidosis is often called *The Great Mimicker* due to its ability to imitate numerous other diseases. It is vital to avoid misdiagnosis and mistreatment, as both can lead to adverse outcomes [8,9].

## Case presentation

A 46-year-old African American male patient with a past medical history of diabetes, asthma, and fatty liver disease presented to the emergency department (ED), with shortness of breath, wheezing, and cough, He also complained of low back pain, which radiated to the left foot and reported a weight loss of 35 pounds over a couple of months associated with night sweats. He mentioned that he was a truck driver and traveled nationwide. He was once caught in a dust storm in Arizona. He is only sexually active with his wife. He reports that he has smoked half a pack of cigarettes per day for 20 years.

In the ED, the patient was saturated 89% on room air and placed on a 4 L nasal cannula. He was treated with antibiotics, which included azithromycin and ceftriaxone, and he also received methyl prednisone 125 mg intravenous (IV) and magnesium sulfate 2 g IV, ipratropium bromide 0.5 mg/albuterol sulfate 3.0 mg nebulizer, and 2 L bolus of normal saline. Computerized tomography with an angiogram of the chest ruled out pulmonary embolism. Still, it showed diffuse ground glass opacification of bilateral lung field and macronodular alveolar opacities and multiple enlarged lymph nodes in the mediastinum and the right hilum. The laboratory results were notable for a lactic acid level of 1.1 mmol/L, a white blood cell count of 15.6 x 10³/µL, and a hemoglobin level of 10 g/dL. The patient was then admitted to the hospital for acute respiratory failure and suspected pneumonia.

The patient had multiple hospitalizations for shortness of breath; he was admitted one month prior for diffuse low back pain, ongoing for three months with chills at night and ambulatory dysfunction. He also reported no muscle weakness, numbness, or tingling sensations. There was no bowel or bladder dysfunction and no saddle anesthesia. He denied IV drug use. He was seen by a doctor six months prior and had a workup with labs that showed an elevated erythrocyte sedimentation rate (ESR) of 145 mm/hour, CRP of 21 mg/L, and unremarkable CT scans of his chest. MRI of the thoracolumbar spine showed nonspecific high signal focus within the central spinal cord at the level of T7, measuring 6 mm x 3 mm. CT cervical and lumbar spine was unremarkable. He reported similar complaints of cervical lymph node enlargements and had developed nonpainful papulonodular lesions over his face for the past few weeks. He was admitted to the hospital and then left against medical advice. 

During this hospitalization, he had intermittent spikes of fever with a maximum temperature of 38.7 °C initially but remained afebrile afterward. He was tachycardic and required a continuous 2 to 3 L of oxygen through a nasal cannula to maintain oxygen saturation above 93 percentiles. Labs were remarkable for leukocytosis with a white blood count of 16 x 10^3^/mcL, normal serum electrolytes, and normal serum calcium, but elevated alkaline phosphatase at 176 mg/dL. Alanine transaminase (ALT) and aspartate aminotransferase (AST) were normal. The HIV combo antigen/antibody test was negative, as were the respiratory panel and COVID-19 test. Inflammatory markers, including ferritin, LDH, and ACE levels were normal, except for elevated CRP and ESR. His brain MRI was remarkable for T2/FLAIR white matter hyperintensity, which was nonspecific. Due to the ongoing back pain and imaging findings, neurology was consulted for possible multiple sclerosis, and a lumbar puncture was performed. Cerebrospinal fluid (CSF) analysis showed 1 lymphocyte/mcL, a glucose level of 72 mg/dL, and normal protein at 20 mg/dL. During the hospitalization, he also complained of left ankle pain without joint redness or effusion in the joint.

Rheumatology was consulted for the diagnosis of sarcoidosis, given bilateral hilar adenopathy, and neurological symptoms with imaging findings of parenchymal lesions, facial pustular lesions, constitutional symptoms, and lung and bone involvement. In the beginning, the patient refused the biopsy procedure. The rheumatologist discussed the imaging findings with the radiologist, who supported the diagnosis of sarcoidosis. However, due to the patient's continued requirement for oxygen while on the medical floor, the physician used clinical judgment to start steroids for three days. Despite the steroids, the patient's symptoms worsened. The patient was upgraded to the ICU as he required continuous high-flow oxygen despite being on steroids and broad-spectrum IV antibiotics for suspected pneumonia

A pulmonologist was consulted, and he eventually underwent bronchoscopy with broncho-alveolar lavage (BAL) and lung biopsy. The bronchial washing culture result was unremarkable, CSF AFB culture was negative. Pathology of lung biopsy days later revealed fungal morphology consistent with Coccidioides species, focal histiocytic aggregate, and multinucleated giant cell formation; the fungal stain supported the diagnosis, and the patient started on IV amphotericin B liposomal 5 mg/kg; then he had improvement in his respiratory symptoms and downgraded to the general floor before discharged home.

**Figure 1:**
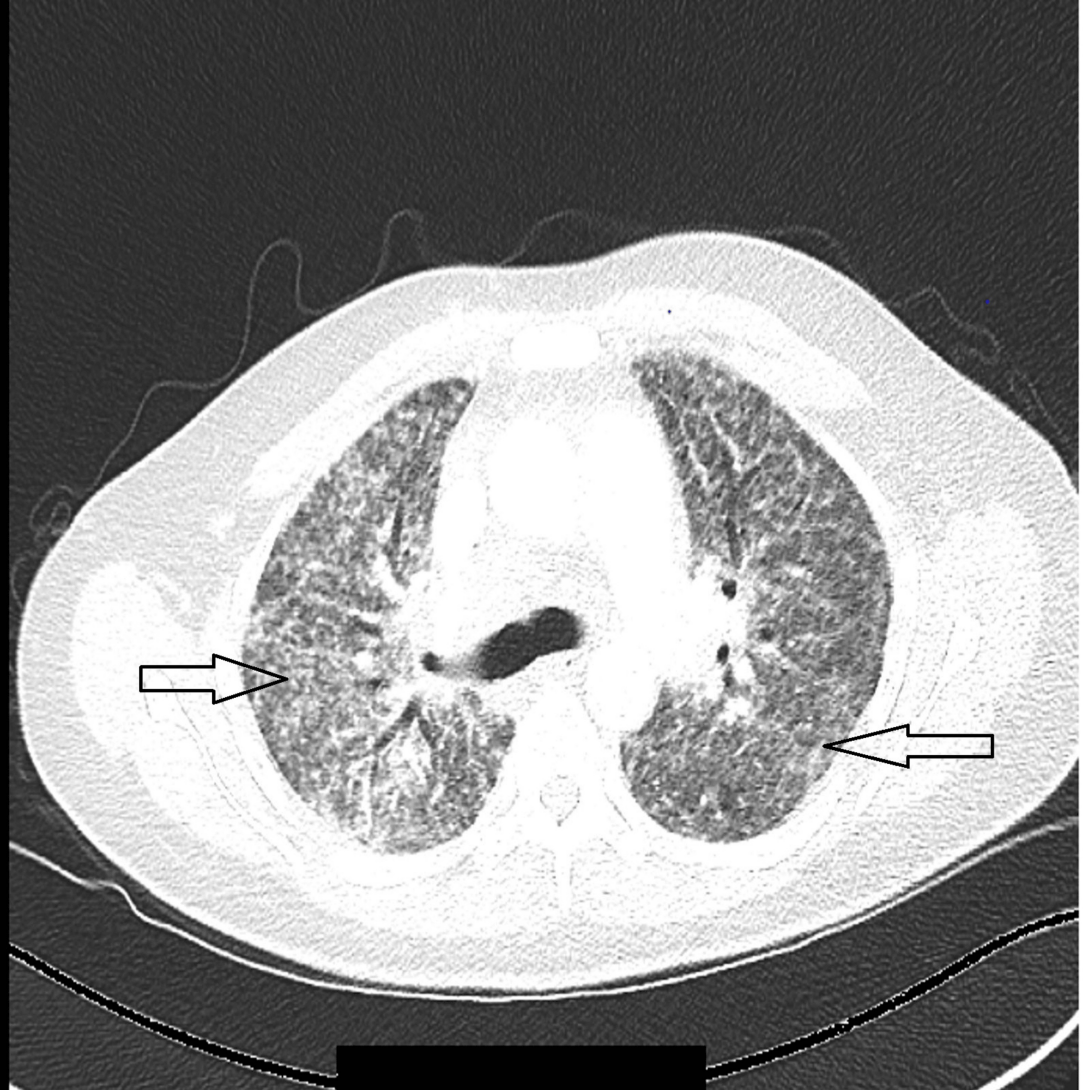
CT scan of the chest, arrows pointed to areas with diffused ground-glass opacification of bilateral lung fields.

**Figure 2:**
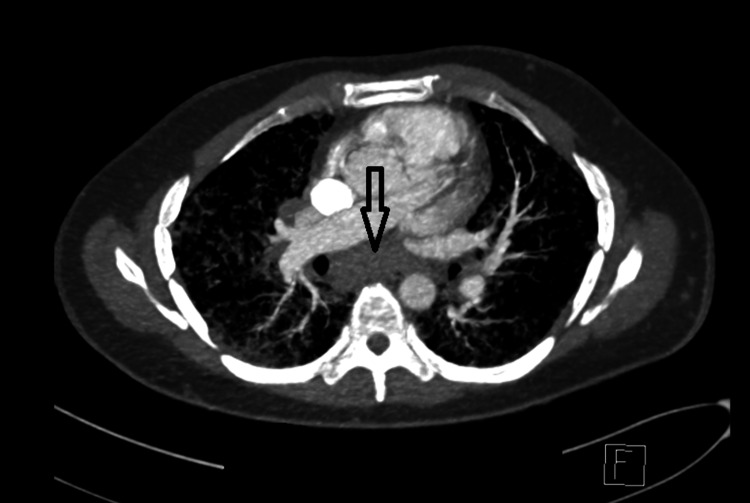
CT scan of the chest, with the arrow pointed to hilar lymphadenopathy.

**Figure 3:**
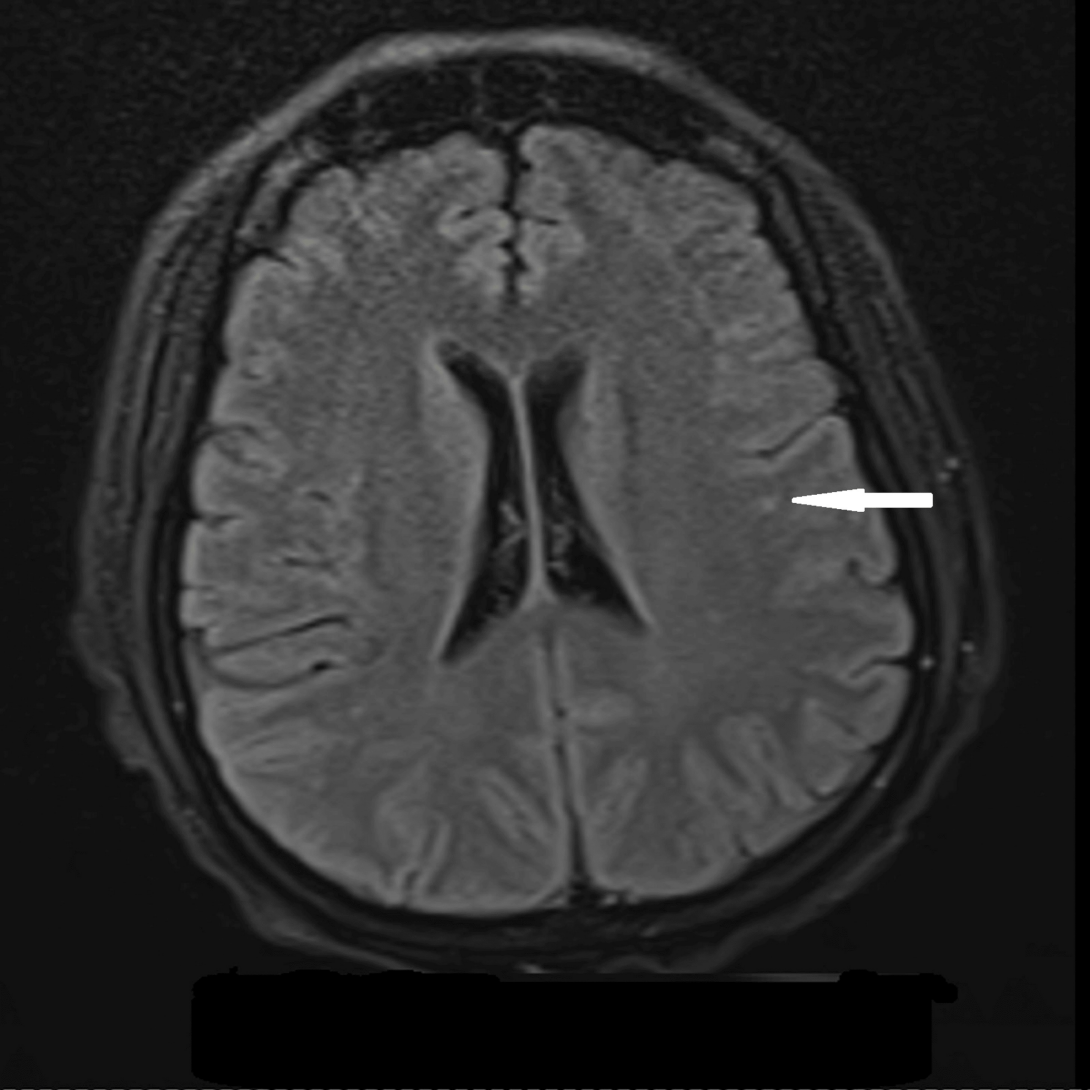
Brain MRI showing T2 hyperintense lesions marked by a white arrow on the subcortical and deep white matter areas.

**Figure 4:**
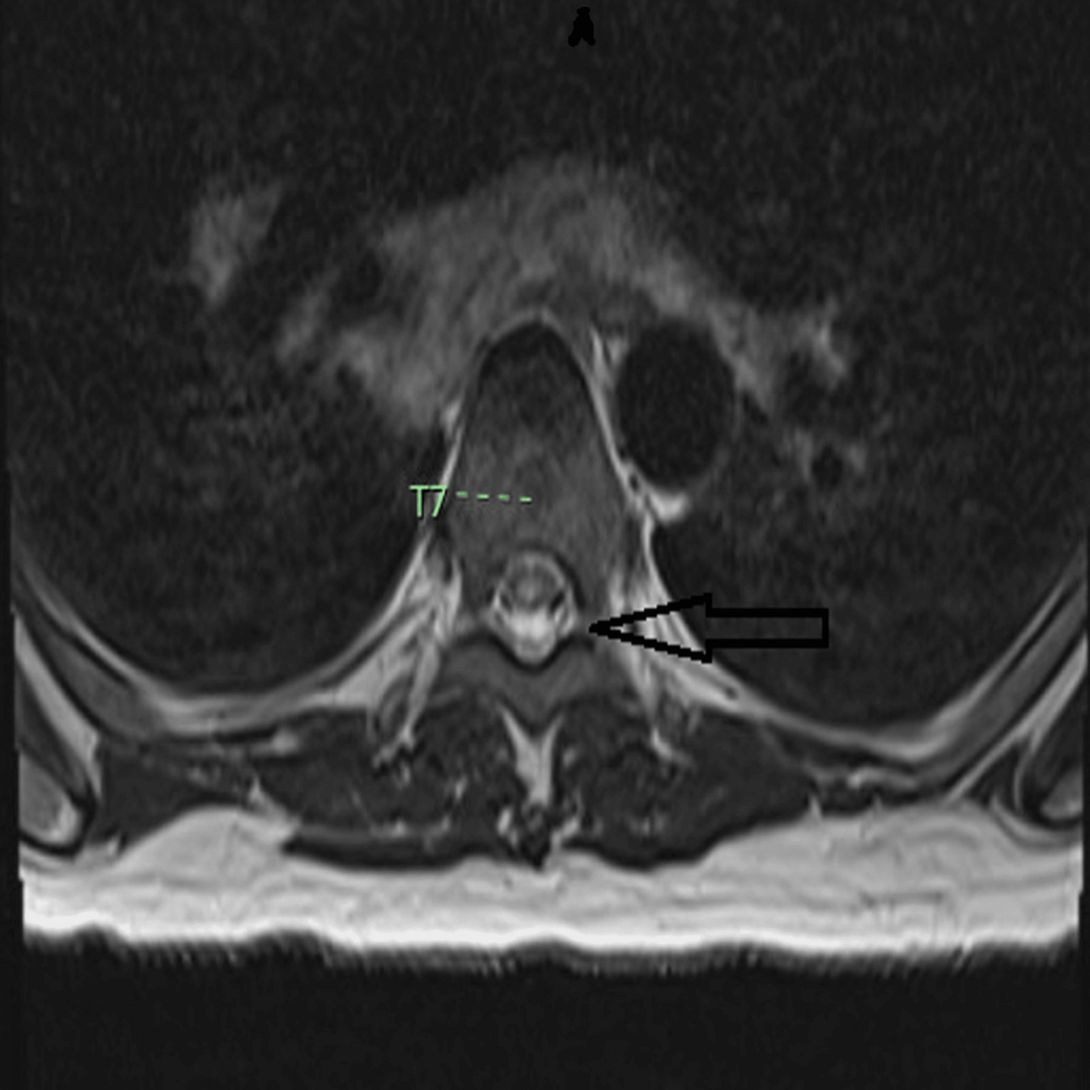
Thoracic spine MRI, T2-weighted image. There is increased signal intensity within the central spinal cord measuring 3 mm x 6 mm at the level of the T7 vertebra.

## Discussion

Sarcoidosis can often resemble other diseases such as lymphoma, tuberculosis, autoimmune diseases like hypersensitivity pneumonitis, fungal infections such as coccidioidomycosis, and other granulomatous diseases. Therefore, thorough investigations and careful analysis are essential to rule out other potential causes and confirm the diagnosis to begin appropriate therapy.

Distinguishing coccidioidomycosis from sarcoidosis or other diseases requires a comprehensive approach involving detailed clinical evaluation, laboratory studies, and imaging techniques, Research reported that the PCR test to detect the Coccidiosis DNA in the patient’s clinical specimens has 98% sensitivity and 100% specificity for the detection of the disease, but unfortunately not available commercially [10]. Also, it is essential to exclude infectious causes of granulomas, as they may mimic the presentation of sarcoidosis. Relying on laboratory studies rather than clinical features is crucial in excluding infectious etiology. Considering the recommended laboratory studies during the patient's evaluation is essential. Table 1 [11] presents the recommended laboratory studies to consider when evaluating the patient.

**Table 1:** Infectious causes of granulomas and the laboratory studies to consider to rule out infection. Note: the table composed from [11].

Infectious causes of granulomas	
Bacteria	Actinomyces, Bartonella, Borrelia burgdorferi, Brucellosis, Mycobacterium tuberculosis, Non-tuberculous Mycobacteria, Nocardia, Q Fever, Treponema pallidum, Whipple's Disease
Fungi	Aspergillosis, Blastomycosis, Candida, Cryptococcus, Histoplasma
Parasitic	Leishmaniasis, Schistosomiasis
Viral	Cytomegalovirus, Epstein-Barr Virus
Laboratory studies to rule out infection	
Histopathologic examination	Ziehl-Neelsen stain or Auramine-rhodamine fluorescence for Mycobacteria, Grocott Methenamine Silver stain for Fungi, TB PCR
Culture	Bacteria (aerobic and anaerobic), Fungi, Mycobacteria
Serology	Histoplasma, Syphilis, Lyme Disease

Chest X-ray findings in pulmonary coccidioidomycosis infection can include interstitial and reticulonodular infiltrates, fibro-cavitary lesions, or mediastinal lymphadenopathy, and these radiological findings can resemble those of sarcoidosis or malignancy [12]. Serological tests for diagnosing coccidioidomycosis have variable sensitivity. In one study of 41 patients with culture-confirmed coccidioidomycosis, the enzyme immunoassay (EIA) to detect Coccidioides antibodies was positive in 34 patients (81%), immunodiffusion complement-fixing (IDCF) was positive in 29 patients (71%), and the complement-fixing antibodies (CF) test was positive in 23 patients (56%), while 6 patients (15%) tested negative for all serological tests [10].

Three criteria must be met to diagnose sarcoidosis: clinical and radiological findings, exclusion of other diseases, and histopathological findings of non-caseating granulomas [7]. The radiological findings of the lungs in disseminated fungal infection may look like those of sarcoidosis. In this particular case, extensive disease was observed in an immunocompetent individual. Despite the patient's well-controlled diabetes and absence of residency in an endemic area, the only pertinent part of the history was his travel to endemic areas for short periods raised the possibility of disseminated fungal infection. The patient's symptoms, initially attributed to sarcoidosis, which later did not respond to steroids, led us to consider other potential causes, including fungal infection.

On the other hand, the diagnosis of coccidioidomycosis, also known as Valley fever, presents a challenge due to its diverse clinical presentations and the similarity of symptoms to other diseases like sarcoidosis. A prompt and accurate diagnosis is essential to avoid inappropriate treatment and poor patient outcomes. The infection varies from showing no symptoms to developing severe disseminated disease, especially in immunocompromised individuals or those with risk factors such as advanced age, diabetes, or immunosuppression [10,13].

Challenging cases like this one require histopathological examination to confirm the presence of fungal elements, such an important step in early identification enables healthcare providers to initiate appropriate antifungal therapy, which leads to significant improvement of patient outcomes and reducing the risk of complications [10,14]. Multiple case reports support the hypothesis that coccidioidomycosis could be a potential trigger for sarcoidosis, especially in endemic areas like Arizona [15], or that there could be a co-occurrence of both sarcoidosis and coccidioidomycosis in a patient, which make the diagnosis more challenging [16].

Since there is an overlap in clinical symptoms and no blood work to make a definite diagnosis, starting the patient with steroids in the case of fungal infection can worsen the situation. Therefore, we recommend that these patients undergo BAL and a lung biopsy to establish a definite diagnosis without delaying their care.

## Conclusions

The case report adeptly discusses the complexities in differentiating coccidioidomycosis from similar conditions, such as sarcoidosis. It underscores both the importance and the challenges of achieving accurate and early diagnosis to minimize morbidity and mortality. This succinct and evidence-based report serves as an essential resource for clinicians, providing critical insights and guidelines for the effective management of this serious infection.
